# The phylogenomic position of the Critically Endangered Largetooth Sawfish *Pristis pristis* (Rhinopristiformes, Pristidae), inferred from the complete mitochondrial genome

**DOI:** 10.1080/23802359.2018.1501315

**Published:** 2018-09-10

**Authors:** Peter M. Kyne, Jun-Jie Wang, Dan Xiang, Xiao Chen, Pierre Feutry

**Affiliations:** a Research Institute for the Environment and Livelihoods, Charles Darwin University, Darwin, Australia;; b Key Laboratory of Tropical and Subtropical Fishery Resource Application and Cultivation, Ministry of Agriculture, Pearl River Fisheries Research Institute of Chinese Academy of Fishery Sciences, Guangzhou, PR China;; c Shantou University Medical College, Guangzhou, PR China;; d College of Marine Sciences, South China Agricultural University, Guangzhou, PR China;; e CSIRO Oceans and Atmosphere, Hobart, Australia

**Keywords:** *Pristis pristis*, mitochondrial genome, Pristidae, threatened species

## Abstract

The complete mitogenome of the Critically Endangered Largetooth Sawfish *Pristis pristis* (Rhinopristiformes, Pristidae) is presented in this study. The genome is 16,912 bp in length with a nucleotide base composition of 32.0% A, 26.5% C, 13.2% G, and 28.3% T, containing 37 genes typical of vertebrates. Two start (GTG and ATG) and two stop (TAG and TAA/T) codons are found in the protein-coding genes. The 22 tRNA genes range from 66 bp (tRNA-Ser2) to 75 bp (tRNA-Leu1). The tRNA-Pro gene is duplicated with an unknown sequence between the two copies. Bayesian phylogenetic reconstruction showed that *P. pristis* clusters with the *Pristis* clade with strong posterior probability (100%).

The sawfishes (Rhinopristiformes, Pristidae) are amongst the world’s most threatened families of fishes, with all five recognized species assessed as Critically Endangered or Endangered on the IUCN Red List of Threatened Species (Dulvy et al. 2016; IUCN 2017). The Largetooth Sawfish *Pristis pristis* (Linnaeus, 1758) was once widespread in inshore tropical regions of the Indo-Pacific and Atlantic Oceans, but its distribution has been severely reduced (Kyne et al. 2013; Dulvy et al. 2016). It is a large (to >6.5 m total length) euryhaline species of tropical rivers, estuaries, and coastal habitats, and significant population declines and range reductions has led to an assessment of Critically Endangered on the IUCN Red List (Kyne et al. 2013). Here, we provide the whole mitochondrial genome of *P. pristis* and use it to infer the phylogenetic position of the species.

A tissue sample (fin clip) was collected from a specimen of *P. pristis* captured and released at Beeboom Crossing (−13.8622°, 131.0744°) in the mid reaches of the Daly River, Northern Territory, Australia, on 10 October 2011. The specimen was an immature male measuring 122 cm total length. The tissue sample was stored at CSIRO, Castray Esplanade, Hobart, Tasmania, Australia, with voucher no. PMD001. The total DNA was extracted from the fin clip. The experimental protocol and data analysis followed Chen et al. (2014). *Pristis pristis*, together with the three other sawfish species with available mitogenomes (Narrow Sawfish *Anoxypristis cuspidata* (Chen et al. 2016a), Dwarf Sawfish *Pristis clavata* (Feutry et al. 2015), and Smalltooth Sawfish *Pristis pectinata* (Chen et al. 2016b)), and eight other chondrichthyan species with complete mitogenomes available in GenBank were used to construct the Bayesian phylogenetic tree. The tree was constructed primarily with available mitogenomes from the order Rhinopristiformes. The GTR + I + G model was selected and three partitions were defined: (1) 12S and 16S rRNA genes; (2) the first; and, (3) second codons of all protein-coding genes except ND6 since this gene was encoded on the light strand.

The *P. pristis* mitogenome (Genbank accession No. MH005928) is 16,912 bp long and its nucleotide composition is as follows: 32.0% A, 26.5% C, 13.2% G, and 28.3% T. The A + T rich and G + C poor pattern is similar to other vertebrates. The gene arrangement and transcriptional orientation in *P. pristis* are identical to most vertebrate mitogenomes, containing 13 protein-coding genes, 22 tRNA genes, 2 rRNA genes, and 1 non-coding control region. There are 32 bp short intergenic spaces located in 13 gene junctions and 23 bp overlaps located in 5 gene junctions.

Except for the CO1 gene, which starts with a GTG codon, the other protein genes start with the standard ATG codon. The ND6 gene has a TAG terminal codon, but the other genes have TAA/T codons. Both the 12S rRNA (968 bp) and the 16S rRNA (1,691 bp) genes are located between tRNA-Phe and tRNA-Leu1 genes, separated by a tRNA-Val gene. The length of the 22 tRNA genes ranges from 66 bp (tRNA-Ser2) to 75 bp (tRNA-Leu1). All tRNA genes except tRNA-Ser2 can fold into a typical clover-leaf secondary structure. Unusually, the tRNA-Pro gene is duplicated and an unknown sequence is present between the two copies. Further investigation is required to understand the role, if any, of this duplicated region. The origin of L-strand replication sequence (34 bp), which can fold a hairpin structure as a signal to initiate the replication of L-strand, was identified between the tRNA-Asn and the tRNA-Cys genes.

All nodes in the Bayesian phylogenetic tree were strongly supported (Figure 1). All four species of the family Pristidae clustered together with the three *Pristis* species (*P. pristis*, *P. clavata*, and *P. pectinata*) forming a monophyletic group. The inclusion of the mitogenome of the fifth sawfish species, the Green Sawfish *Pristis zijsron*, in the phylogenetic tree would assist in fully resolving the phylogeny of this family.

**Figure 1.**
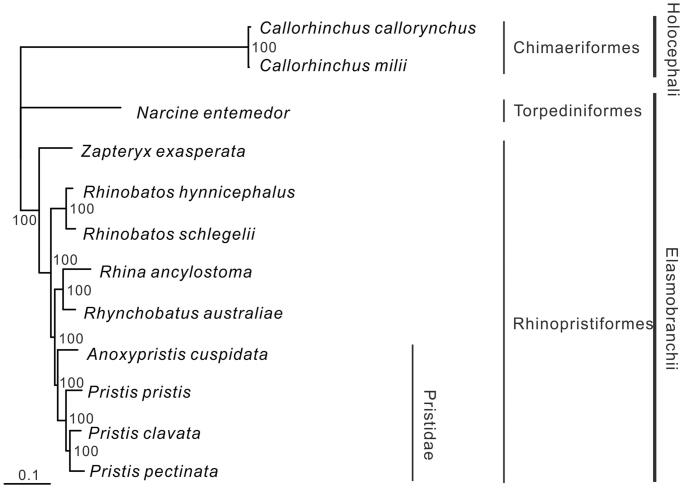
Phylogenetic position of *Pristis pristis*. The holocephalans *Callorhinchus callorynchus* (NC_014281.1) and *Callorhinchus milii* (NC_014285.1) were selected as the outgroup. Other species are Torpediniformes: *Narcine entemedor* (NC_025512.1); and Rhinopristiformes: *Zapteryx exasperata* (NC_024937.1), *Rhinobatos hynnicephalus* (NC_022841.1), *Rhinobatos schlegelii* (NC_023951.1), *Rhina ancylostoma* (NC_030215.1), *Rhynchobatus australiae* (NC_030254.1), *Anoxypristis cuspidata* (NC_026307.1), *Pristis clavata* (NC_022821.1), and *Pristis pectinata* (NC_027182.1).
